# Hydrophobic Capillary Ceramic-Membrane Contactor for Recovering Ammonia from Sludge Hydrolysate

**DOI:** 10.3390/membranes16040140

**Published:** 2026-04-01

**Authors:** Shiji Sun, Mengfei Liu, Dawei Gong, Kaiyun Fu, Xianfu Chen, Minghui Qiu, Ping Luo

**Affiliations:** 1 College of Environmental Science and Engineering, Nanjing Tech University, Nanjing 211816, China; 2 College of Chemical Engineering, Nanjing Tech University, Nanjing 211816, China

**Keywords:** capillary ceramic-membrane contactors, ammonia recovery, sludge hydrolysate, membrane fouling

## Abstract

Efficient recovery of ammonia from sludge hydrolysate (SH) remains a challenging task. This study developed a superhydrophobic capillary ceramic-membrane contactors (MCs), which, by establishing a stable gas-phase mass transfer interface, provides a reliable guarantee for ammonia recovery under high-temperature, high-pH, and high-organic-load conditions. In a controllable simulation system, the system investigated the effects of key operational parameters such as pH, flow rate, and feed ammonia concentration on ammonia mass transfer behavior, and verified the feasibility of this MCs in efficient ammonia removal. Then, this membrane contactor was applied to the actual sludge hydrolysate (SH) system, and its anti-pollution effects, wetting stability, and adaptability to fluctuating conditions under long-term continuous operation were evaluated. The results showed that after operating for 10 h, the ammonia removal in the simulation system and the actual system reached 93.6% and 90.3%, respectively. During long-term operation, the ammonia recovery reached 90.3%. Meanwhile, the organic matter in SH was completely retained, and (NH_4_)_2_SO_4_ was not contaminated by organic matter. Throughout the entire operation process, the contact angle of the membrane remained above 129.6°. This study provides a theoretical basis and practical reference for recovering ammonia using a hydrophobic capillary ceramic-membrane contactor in SH.

## 1. Introduction

Sludge is the main by-product generated during the wastewater treatment process and it is an important object that urgently needs to be reduced in volume, made harmless, and utilized for resource recovery [1,2,3]. In recent years, the thermal hydrolysis process (THP) has been widely applied to enhance the biodegradability and gas production efficiency of sludge anaerobic digestion (AD) [4,5,6]. However, the THP significantly increases the ammonia concentration in the sludge hydrolysate (SH) [7]. When this SH is reused in the AD system as a liquid-phase carbon source, the excessive ammonia has a toxic inhibitory effect on methanogens, leading to a decrease in methane production and even system instability. This contradiction has become a key issue restricting the coupled application of THP and AD [8,9]. Therefore, recovering ammonia from SH is not only a necessary condition for ensuring the stable operation of AD, but also an important way to achieve the recycling and utilization of ammonia resources.

**Figure 1:**
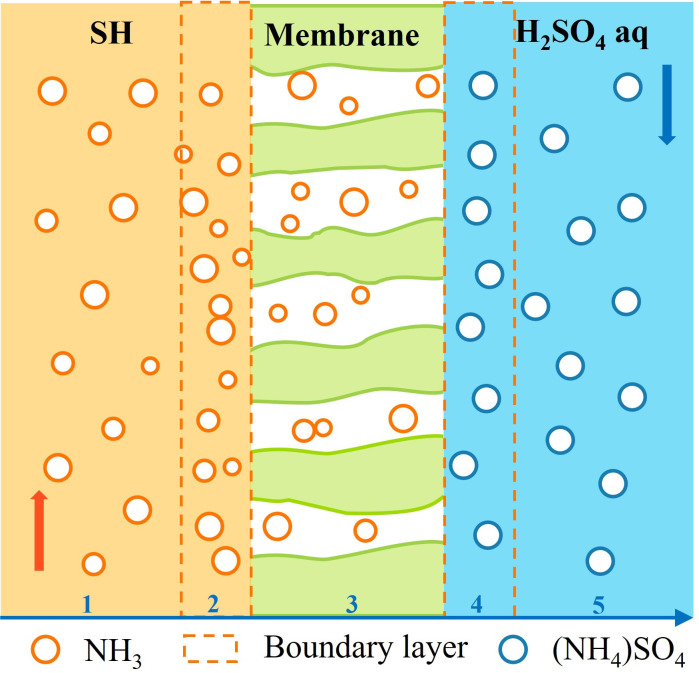
Mechanism of ammonia mass transfer by membrane contactors (From 1 to 5).

At present, the main technologies for recovering ammonia from the liquid phase include steam-stripping [10], adsorption [11], ion exchange, and reverse osmosis [12]. In recent years, membrane contactors (MCs) have attracted extensive attention due to their high mass transfer efficiency, ability to achieve selective separation, and direct production of liquid fertilizers such as (NH_4_)_2_SO_4_ [13,14]. The overall mass transfer process consists of five main steps [15,16,17]: (1) diffusion of ammonia from the SH to the boundary layer near the membrane surface; (2) transfer of ammonia across the boundary layer; (3) transport of ammonia through the gas-filled membrane pores; (4) reaction of ammonia with the absorbent; (5) diffusion of the resulting ammonium salt across the boundary layer into the absorbent solution, as illustrated in Figure 1. The core advantages of this technology lie in the formation of a stable liquid–liquid interface through hydrophobic nanoporous membranes. Under the driving force of concentration gradients and partial pressure differences, free ammonia is transferred across the membrane in gaseous form and captured by acidic absorbent solutions [18,19,20]. This process avoids gas escape and phase dispersion issues, and the flow rates of the fluids on both sides can be independently controlled, effectively preventing foaming and flooding [20]. Additionally, the MC technology features low energy consumption, stable operation, and a small carbon footprint, making it more sustainable compared to traditional methods.

Currently, most MCs use hydrophobic-polymer membrane materials (such as PTFE, PVDF, or PP) [21,22,23] as the interface between the gas and liquid phases. However, the high organic load, high alkalinity, high temperature, and complex composition of the SH make traditional polymer membranes prone to contamination, scaling, wetting, and chemical degradation during ammonia recovery, thereby limiting their long-term stable operation and efficient ammonia recovery, therefore, it is extremely important to develop a membrane that can withstand harsh conditions [14,24,25]. In contrast, hydrophobic capillary ceramic membranes have excellent thermal stability, chemical tolerance, and mechanical strength, and their surfaces are smooth and pore diameters are uniform, which can maintain a non-wetting state under harsh conditions [26].

It is precisely due to the excellent structural stability and chemical tolerance of ceramic membranes that ceramic MCs have been widely applied in various separation and recovery scenarios. For instance, Mohammad et al. [27] used zirconia and kaolin as raw materials and modified them with fluorinated alkyl silanes (FAS) for grafting to prepare hydrophobic hollow-fiber ceramic membranes, achieving 90% ammonia recovery from wastewater; Xu et al. [28] used hexadecyltrimethoxysilane to perform hydrophobic modification on Al_2_O_3_ membranes and used them to absorb CO_2_ in the air, obtaining a high flux of 4.83 mol·m^−3^·s^−1^. Although ceramic MCs perform outstandingly in other systems, there is still a lack of research on their application in actual SH for ammonia recovery.

Considering the complex composition and high fouling risk of the SH system, this study developed a superhydrophobic capillary ceramic-membrane contactor for efficient ammonia recovery. It systematically evaluated its mass transfer performance and operational stability. To clarify the applicability of the membrane contactor in complex systems, a NH_4_Cl solution was used as a control simulation system. The effects of pH, ammonia concentration, and flow rate on ammonia mass transfer behavior were systematically investigated to reveal the basic mass transfer laws. Subsequently, this system was applied to the actual SH, with a focus on evaluating its denitrification efficiency and long-term operational stability, as well as its membrane fouling and wetting behavior under high-organic-load and multi-component-coexistence conditions.

## 2. Materials and Methods

### 2.1. Chemicals and Materials

The main reagents used in the experiment include NH_4_Cl (Xilong Science Co., Ltd., Shantou, China, purity > 99%), H_2_SO_4_ (Shanghai Lingfeng Chemical Reagent Co., Ltd., Shanghai, China, purity > 98%), NaOH (Shanghai Wokai Biotechnology Co., Ltd., Shanghai, China, purity > 99%), hexadecyltrimethoxysilane (Shanghai Auding Biochemical Technology Co., Ltd., Shanghai, China, GC grade, ≥85%), and anhydrous ethanol (Wuxi Yasheng Chemical Co., Ltd., Wuxi, China. analytical grade.). All aqueous solutions were made with deionized water (with a conductivity < 5 μs·cm^−1^). The waste-activated sludge was collected from the Ge Tang Sewage Treatment Plant in Nanjing, Jiangsu Province, China.

The hydrophobic capillary ceramic membrane (Nanjing Jiusi High-tech Co., Ltd., Nanjing, China) used in this study has a pore size of 100 nm and a porosity of 40%. Table 1 summarizes the detailed characteristics of these ceramic membranes and membrane contactor modules. The hydrophobic modification was carried out using an ethanol solution of 3 g/L hexadecyltrimethoxysilane, and the pH of the solution was adjusted to 1 with nitric acid. After the hydrophilic ceramic membrane was immersed in the solution for 24 h, it was taken out and dried to obtain the hydrophobic membrane [29,30].

The sludge is sieved through an 80-mesh screen and mixed with deionized water, adjusted to a moisture content of 90%. Then it is added to a high-pressure reactor (Yantu Scientific Instrument Co., Ltd., Shanghai, China), heated to 180 °C for flash evaporation, and the resulting sludge solution is obtained. The sludge solution is centrifuged at 8000 rpm for 10 min, and the supernatant is collected for subsequent ammonia recovery experiments. To evaluate the applicability of the ceramic MC to SH with different ammonia concentrations, two batches of SH were prepared. They were designated as 1411 N and 554 N according to their ammonia concentrations. The specific parameters are listed in Table 2.

### 2.2. Experimental Setup

**Figure 2:**
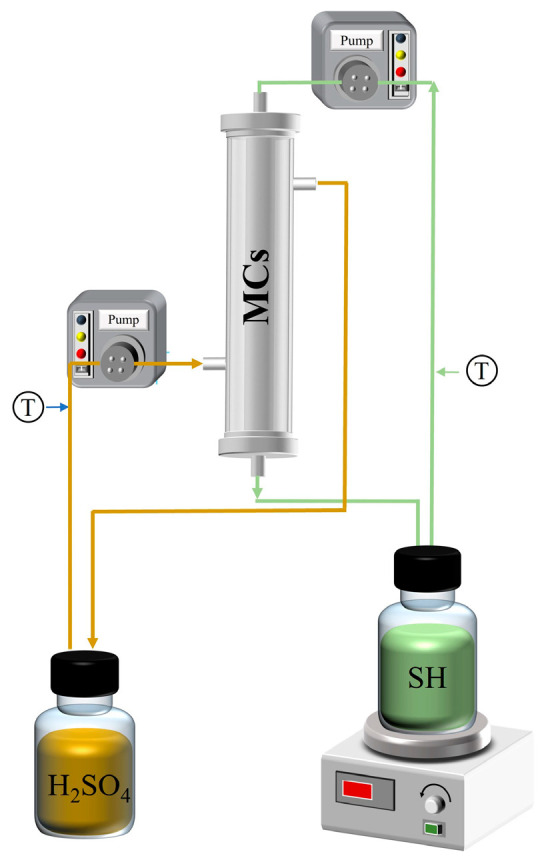
Membrane absorption experimental-device diagram.

The prepared absorption liquid (150 mL) and SH (150 mL) were, respectively, placed in the reactor chamber (250 mL). Using a peristaltic pump (produced by BaodingChuangrui Precision Pump Co., Ltd., Baoding, China), SH and the absorption liquid were pumped in opposite directions at a constant flow rate into the MC tube side and the shell side, as shown in Figure 2. This ensures that the two phases have sufficient contact, facilitating ammonia mass transfer. During the experiment, samples were taken from the SH and absorbent-solution feed tanks. The collected samples were stored in the refrigerator. If the analysis could not be completed within 24 h, the hydrolysate was acidified to ensure the accuracy of the measurement.

First, NH_4_Cl solution was used to simulate SH to investigate the effects of various factors on ammonia removal efficiency and ammonia flux: pH (9–12), temperature (50 °C), flow rate (0.03–0.12 m/s), and ammonia concentration (500–2000 mg/L). The test period lasted for 10 h, with samples being taken once every hour.

Subsequently, two types of SH (1411 N and 554 N) were added to the feed tank, and each batch was processed for 10 h. The three batches of SH were, respectively, named 1–1411 N, 2–1411 N, and 3–554 N according to the processing sequence. Each time the SH was replaced, the MC was flushed with clean water for 5 min, and the absorption liquid was not replaced. Throughout the entire long-term operation process, systematic evaluations were conducted on the removal efficiency of ammonia, the mass transfer coefficient, flux, recovery performance, and the changes in membrane contamination characteristics.

### 2.3. Analysis and Characterization Methods

#### 2.3.1. Analysis of Sludge Hydrolysate

The pH was measured using the PHS-3C Leici pH meter (Shanghai Leici Instrument Co., Ltd., Shanghai, China). COD was analyzed using the Hach high-range COD test kit (Hash Water Quality Analysis Instrument (Shanghai) Co., Ltd., Shanghai, China, 200–15,000 mg/L) and the Hach DR1900 spectrophotometer (Hash Water Quality Analysis Instrument (Shanghai) Co., Ltd., Shanghai, China). Total carbohydrates were determined using the modified phenol–sulfuric acid method, with glucose as the standard solution. The ammonia nitrogen was determined using a Kjeldahl nitrogen (K9840, Shandong Haineng Instrument Co., Ltd., Jinan, China, detection limit 0.1 mg/L, relative standard deviation < 1.5%) analyzer.

#### 2.3.2. Membrane Characterization Method

The surface morphology and roughness of the membrane were analyzed using field emission scanning electron microscopy (Hitachi S-4800, Hitachi Limited, Tokyo, Japan) and atomic force microscopy (Dimension Icon, Bruker Corporation, Karlsruhe, Germany), respectively. The changes in functional groups of the membrane before and after hydrophobic modification were characterized by FTIR (Nicolet iS20, Thermo Fisher Scientific, Waltham, MA, USA). The hydrophobic modification of the membrane was evaluated by dynamic water droplet contact angle measurement (OCA25, Dataphysics, Stuttgart City, Germany), with 1 μL deionized water being dropped at 6 different positions on the membrane surface for testing [31].

### 2.4. Evaluation of Membrane Absorption Performance

The ammonia removal efficiency can be expressed by the following formula:

(1) $$NH_{3}\;\mathrm{removal}\;(\%)=\frac{C_{0}-C_{t}}{C_{0}}\times 100$$

In the equation, *C*_0_ is the initial ammonia concentration in the SH, mg/L; *C_t_* is the ammonia concentration in the SH at the sampling time point, mg/L. The recovery efficiency of ammonia can be expressed by the following formula:

(2) $$NH_{3}\;\mathrm{recovery}\;(\%)=\frac{S_{t}-S_{0}}{C_{0}-C_{t}}\times 100$$

In the equation, *S_t_* is the ammonia concentration in the absorbent solution at the sampling time point, mg/L. *S*_0_ is the initial ammonia concentration in the absorbent solution, mg/L. The mass transfer coefficient during the experimental process can be calculated using the following equation:

(3) $$K=\frac{V_{t}}{A_{m}t}\ln\frac{C_{0}}{C_{t}}$$

In the equation, *K* is the overall mass transfer coefficient, m·s^−1^; *V_t_* is the initial volume of the SH, m^3^; *A_m_* is the membrane area, m^2^; and *t* is the operation time, s. The ammonia flux can be expressed by the following equation:

(4) $$J_{A}=\frac{\Delta m}{A_{m}\Delta t}$$

In the equation, Δ*m* is the mass of ammonia transferred from the tube side to the shell side, kg; Δ*t* is the experimental operation time, h.

## 3. Results and Discussion

### 3.1. Membrane Characterization

**Figure 3:**
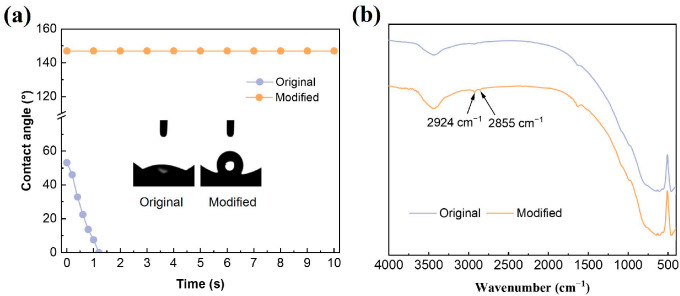
Dynamic water contact angles (**a**) and FTIR spectra (**b**) of the ceramic membranes before and after surface modification.

To characterize the hydrophobic surface, the dynamic water contact angle was first measured (Figure 3a). The original ceramic membrane exhibited a rapid decrease in water contact angle from 52° to 0°, confirming its strong hydrophilicity due to the abundance of surface hydroxyl groups. In contrast, the modified membrane showed a significantly increased water contact angle of 142° and remained stable during the measurement, demonstrating excellent hydrophobicity. FTIR analysis further revealed two new peaks at 2924 cm^−1^ and 2855 cm^−1^ (Figure 3b), which are assigned to the asymmetric and symmetric stretching vibrations of C-H bonds, indicating that the modifier was successfully grafted onto the ceramic-membrane surface. Based on the above analyses, this remarkable increase in hydrophobicity effectively establishes a high-interfacial-energy barrier that prevents aqueous liquid intrusion (wastewater or acidic solution), thereby maintaining gas-filled pores that are essential for ammonia volatilization and diffusion.

**Figure 4:**
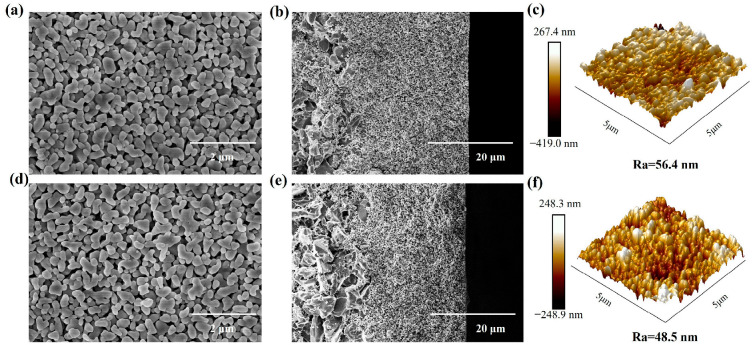
Surface characterization of membranes before and after modification. SEM and AFM images before membrane modification (**a**–**c**). SEM and AFM images after membrane modification (**d**–**f**).

The surface, cross-section, and surface roughness of the original membrane and the modified membrane were measured by SEM and AFM, as shown in Figure 4. No obvious changes were observed in the SEM and AFM images of the membrane before and after hydrophobic modification, and the influence of hydrophobic modification on the microstructure of the membrane was not significant.

### 3.2. Influencing Factors of Ammonia Removal

During the ammonia recovery process in MC, various factors such as temperature, pH, flow rate, and ammonia concentration all have significant impacts on the removal of ammonia. Since the SH temperature is generally around 50 °C, in order to better simulate the actual application conditions, this study fixed the feed temperature at 50 °C and focused on investigating the effects of pH, flow rate, and ammonia concentration on the ammonia removal efficiency.

**Figure 5:**
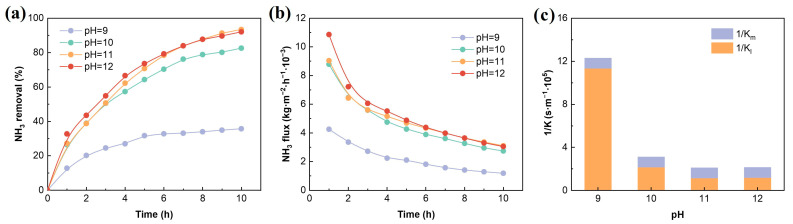
The influence of pH on ammonia removal (**a**), mass transfer flux (**b**), and mass transfer resistance (**c**). Experimental conditions: flow rate = 0.12 m/s, ammonia concentration = 500 mg/L, temperature = 50 °C, H_2_SO_4_ concentration = 0.4 mol/L.

SH is usually neutral, so alkaline substances need to be added to adjust the pH and convert the ionized ammonia into free ammonia [28]. Therefore, the feed pH is one of the key factors affecting the efficiency of ammonia removal. As shown in Figure 5a,b, when the pH increases from 9 to 11, the ammonia removal increases from 35.7% to 93%, and the ammonia flux increases from 0.001 to 0.003 kg·m^−2^·h^−1^, increasing by approximately three times. However, when the pH continues to rise to 12, the removal and flux do not show significant improvement. This is because the increase in pH causes the ammonium ions to lose protons and form free ammonia. When the pH increases from 9 to 11, the proportion of free ammonia increases from 63.9% to 99.4%, significantly enhancing the mass transfer driving force. However, when the pH exceeds 11, the concentration of free ammonia is already high enough, and the influence of pH on overall mass transfer tends to weaken. The resistance distribution (The calculation method is shown in Supplementary Materials (S1) shown in Figure 5c further indicates that as the pH increases, the mass transfer resistance on the feed side (K_l_) decreases.

**Figure 6:**
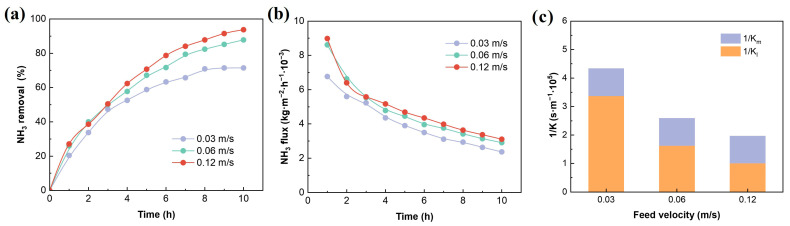
The influence of feed velocity on ammonia removal (**a**), mass transfer flux (**b**), and mass transfer resistance (**c**). Experimental conditions: pH = 11, ammonia concentration = 500 mg/L, temperature = 50 °C, H_2_SO_4_ concentration = 0.4 mol/L.

Further studies were conducted on the influence of SH flow rate on ammonia removal and mass transfer performance. As shown in Figure 6a,b, when the feed flow rate increased from 0.03 m/s to 0.12 m/s, the ammonia removal rose from 71.4% to 93.7%, an increase of 22.3%, the ammonia flux increased from 0.002 to 0.003 kg·m^−2^·h^−1^, and the mass transfer resistance on the feed side (K_l_) also decreased significantly. The results indicate that with the increase in flow rate, the removal efficiency significantly improves. The reason is that under low-flow conditions, the SH is in a laminar-flow state, and the material transfer is mainly controlled by molecular diffusion. The boundary layer is thicker, and the resistance is greater. On the contrary, in high-flow conditions, the fluid turbulence increases, the boundary layer becomes thinner, and the liquid-film resistance decreases, thereby significantly improving the overall material transfer efficiency.

**Figure 7:**
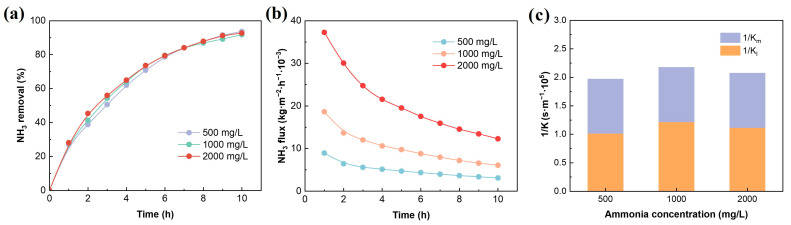
The influence of ammonia concentration on ammonia removal (**a**), mass transfer flux (**b**), and mass transfer resistance (**c**). Experimental conditions: pH = 11, flow rate = 0.12 m/s, temperature = 50 °C, H_2_SO_4_ concentration = 0.4 mol/L.

The ammonia concentration in SH is an important factor affecting ammonia removal. As shown in Figure 7a–c, during continuous operation for 10 h, when the initial concentrations were 500, 1000, and 2000 mg/L, the ammonia removal was 93.6%, 91.8%, and 92.7%, respectively, and the corresponding ammonia fluxes were 0.003, 0.006, and 0.012 kg·m^−2^·h^−1^. The results indicate that although the feed ammonia concentration increased by four times, the removal rate and mass transfer resistance remained stable, indicating that the MC has good adaptability and stable ammonia removal performance over a wide concentration range.

### 3.3. Long-Term Operational Performance

Based on the experimental results in Section 3.2, the hydrophobic capillary ceramic membrane exhibited excellent ammonia removal performance in the NH_4_Cl solution. To further evaluate its feasibility for practical application in the actual SH, experiments were conducted under the optimal operating conditions determined in Section 3.2 (pH = 11, v = 0.12 m/s, T = 50 °C, H_2_SO_4_ concentration = 0.4 mol/L). During the actual processing, the ammonia concentration in SH fluctuated significantly. Therefore, two different ammonia concentrations of SH (1411 N and 554 N) were selected and continuously fed in three batches to evaluate the adaptability of MC to different feeding systems under long-term operation conditions.

**Figure 8:**
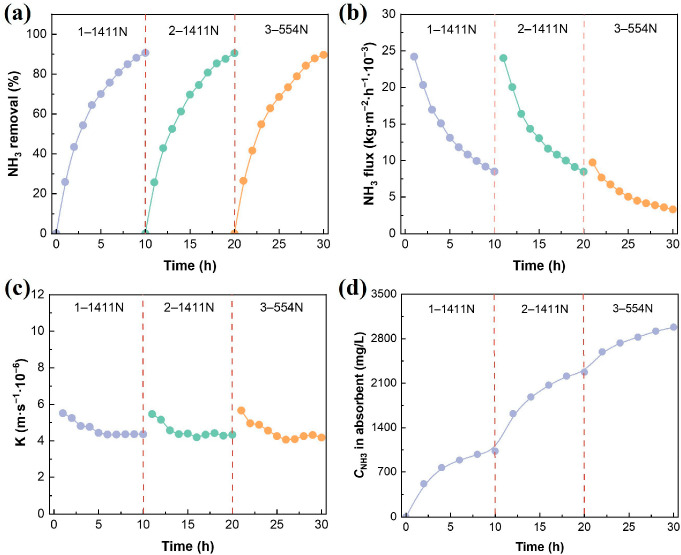
Stability of mass transfer in continuous operation of membrane contactors. Ammonia removal (**a**), ammonia flux (**b**), ammonia mass transfer coefficient (**c**), ammonia recovery performance (**d**). Experimental conditions: pH = 11, flow rate = 0.12 m/s, ammonia concentration = 554–1411 mg/L, temperature = 50 °C, H_2_SO_4_ concentration = 0.4 mol/L.

The stability results are shown in Figure 8a. Even when the feed composition changes, the ammonia removal performance of the MC remains stable. For the three batches of feed (1–1411 N, 2–1411 N, and 3–554 N), after running for 10 h, the ammonia removal reached 90.6%, 90.5%, and 89.6%, respectively, showing only a slight decrease, but still maintaining at approximately 90% overall. The ammonia concentration in the post-treatment SH was significantly lower than the feed requirements for AD. It is worth noting that within 4 h after each batch of SH was added, the ammonia removal was above 61%. However, the remaining ammonia needed 6 h to be removed, which might be related to the decrease in pH value during the operation, resulting in a weakened mass transfer driving force.

The ammonia flux within the MCs serves as a critical metric for assessing deamination performance. As illustrated in Figure 8b, there is a progressive decline in flux over the course of operation. Following the introduction of three distinct SH batches (1–1411 N, 2–1411 N, and 3–554 N), initial hourly flux rates were recorded at 0.024, 0.024, and 0.009 kg·m^−2^·h^−1^, respectively. Notably, the flux transitioned from a sharp reduction to a more gradual decline after approximately 4 h, primarily because over 60% of the total ammonia had been eliminated during that initial period. The residual ammonia concentration significantly decreased, and the partition coefficient at the gas–liquid interface and the transmembrane mass transfer driving force subsequently weakened. After adding the fresh SH for 4 h, the ammonia mass transfer coefficient showed a significant decrease, as shown in Figure 8c. The possible reason is that the ammonia mass transfer driving force decreases as the ammonia concentration decreases, making it difficult to overcome the current resistance quickly, resulting in a decrease in the mass transfer coefficient; the results compared with the polymer membranes are shown in Table 3. After prolonged operation, the spot-like foulants deposited on the membrane surface may block surface pores, leading to a reduction in flux and mass transfer coefficients.

The MCs can directly obtain (NH_4_)_2_SO_4_ while recovering ammonia, without causing the loss of organic substances in the SH. This is a significant advantage of this technology compared to other ammonia recovery methods. As shown in Figure 8d, during the continuous operation for 30 h, the ammonia concentration in the absorption liquid continued to increase and finally reached 2981.47 mg/L. Meanwhile, the ammonia recovery remained at 90.3%. One potential reason for the ammonia recovery effect being lower than the theoretical value is that during the pH adjustment process of SH, some ammonia volatilizes and escapes in the gaseous form. Table 4 shows that no organic substances were detected infiltrating the ammonium sulfate solution throughout the recovery process, and the COD of the two types of SH after treatment did not show a significant decrease, indicating that their organic components were not damaged. In conclusion, the hydrophobic capillary ceramic-MCs not only can efficiently recover ammonia and obtain (NH_4_)_2_SO_4_, but can also maintain the integrity of the organic components in the SH, demonstrating excellent selectivity and operational stability.

### 3.4. Membrane Stability

**Figure 9:**
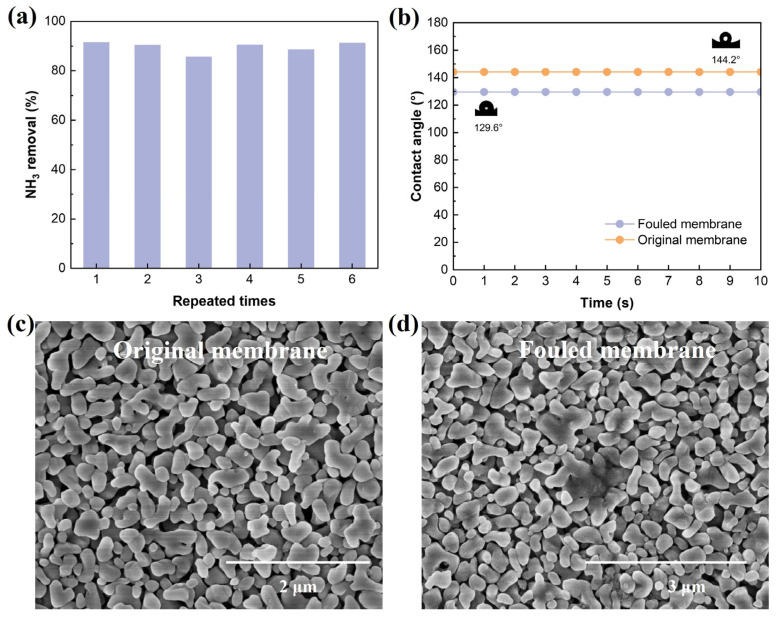
Membrane stability. Stability of ammonia removal (**a**), change in contact angle (**b**), changes in membrane surface (**c**,**d**). Experimental conditions: pH = 11, flow rate = 0.12 m/s, ammonia concentration = 554 mg/L, temperature = 50 °C, H_2_SO_4_ concentration = 0.4 mol/L.

The stability of the membrane is the key factor determining whether the MCs can maintain stable deamination over a long period of time. Under the optimal conditions, the 544 N SH was subjected to 10 h cyclic treatment, and the experiment was repeated six times to verify its stability. As shown in Figure 9a, the ammonia removal of the six experiments remained within the range of 90 ± 2%, indicating that this process has good repeatability and stability.

As shown in Figure 9b, after long-term operation, the water contact angle on the membrane surface slightly decreased, but remained above 129°, indicating that the gas-phase interface within the membrane pores was effectively maintained and no significant wetting phenomenon occurred. The slight reduction in the contact angle might be related to the local attachment of organic substances in SH on the membrane surface, but it did not have a substantial impact on the overall hydrophobic performance of the membrane.

The further morphological analysis results are shown in Figure 9c,d. Before operation, the membrane surface presented a dense and uniformly distributed porous structure; after long-term operation, only local speckled deposits appeared on the membrane surface, which was speculated to mainly originate from the adsorption of organic substances (such as proteins) in SH.

In conclusion, the prepared superhydrophobic capillary ceramic membrane still exhibits excellent anti-wetting ability and operational stability in the complex SH system, providing reliable support for the long-term and stable ammonia recovery achieved by the membrane contactor under actual engineering conditions.

## 4. Conclusions

This study is the first to report the application of superhydrophobic capillary ceramic membranes in the long-term recovery of ammonia from SH. The ammonia recovery efficiency and the long-term operational stability of the membranes were evaluated. The results showed that during long-term continuous operation, the change in ammonia concentration in the feed did not affect the recovery effect, and the ammonia recovery reached 90.3%. The generated (NH_4_)_2_SO_4_ was not contaminated by organic matter. The organic matter in SH was not lost and could be used as a carbon source for anaerobic digestion. After long-term operation, the contact angle of the membrane remained above 129°, and no severe membrane contamination occurred. In conclusion, this superhydrophobic capillary ceramic-MC, as an efficient and stable ammonia recovery technology, provides an important technical approach and application potential for the resource utilization of ammonia in SH.

## Figures and Tables

**Table 1:** Membrane and membrane module characteristics.

	Unit	Value
Membrane		
Outside diameter	mm	6
Inside diameter	mm	4
Effective length	mm	180
Membrane area	m^2^	0.0023
Membrane modules		
Outside diameter	mm	18
Inside diameter	mm	14
Length	mm	340

**Table 2:** Sludge hydrolysate parameters.

Parameters	Unit	1411 N	554 N
NH_3_-N	mg/L	1411	554
COD	mg/L	21,780	17,820
Carbohydrates	mg/L	2960	1740
pH	-	6.8	6.5

**Table 3:** Compared with polymer membrane contactors.

Membrane Types	Pore Diameter (nm)	Feed Solution	Ammonium Concentration (mg/L)	K (m·s^−1^)	Ref
PMP	50	Synthetic wastewater	5000	2.4 × 10^−7^	[17]
PP	250	Human hydrolysis urine	6078	2.6 × 10^−7^	[32]
PTFE	380	Urban wastewater	2000	3.5 × 10^−6^	[23]
PP	30	Urban wastewater	4000	6.5 × 10^−7^	[33]
Ceramics	100	SH	554–1411	4.6 × 10^−6^	This work

**Table 4:** Comparison of SH parameters before and after processing.

Parameters	Unit	1411 N	A-1411 N	554 N	A-554 N
NH_3_-N	mg/L	1411	134.19	554	57.39
COD	mg/L	21,780	19,540	17,820	17,120
Carbohydrates	mg/L	2960	2720	1740	1580

Note: A-1411 N and A-554 N are processed SH.

## Data Availability

The original contributions presented in the study are included in the article. Further inquiries can be directed to the corresponding author.

## Supplementary Materials

The following supporting information can be downloaded at: https://www.mdpi.com/article/10.3390/membranes16040140/s1, S1: Mass transfer resistance calculation.
