# Supplementary material for: Hydrophobic Capillary Ceramic-Membrane Contactor for Recovering Ammonia from Sludge Hydrolysate
Source: Membranes (Basel). 2026 Apr 1;16(4):140. doi: 10.3390/membranes16040140 (PMC13117203; doi:10.3390/membranes16040140)
Supplement: Supplementary file 1 [file membranes-16-00140-s001.zip › membranes-4045633-supplementary.pdf]

Supplementary Materials

Hydrophobic Capillary Ceramic-Membrane Contactor for Recovering Ammonia from Sludge Hydrolysate

Shiji Sun [1], Mengfei Liu [2], Dawei Gong [2], Kaiyun Fu [2], Xianfu Chen [2], Minghui Qiu [2] and Ping Luo [1,*].

1 College of Environmental Science and Engineering, Nanjing Tech University, Nanjing 211816, China. ssj@njtech.edu.cn(S.S.).
2 College of Chemical Engineering, Nanjing Tech University, Nanjing 211816, China; liumengfei@njtech.edu.cn(M.L.); gongdawei@njtech.edu.cn(D.G.); fukaiyun@njtech.edu.cn(K.F.); chenxianfu@njtech.edu.cn (X.C.); qiumh_1201@njtech.edu.cn (M.Q.).
*Correspondence: luoping@njtech.edu.cn (P.L.).

S1. Mass transfer resistance calculation

Overall mass transfer resistance(1/K):

$$\frac{1}{K}=(\frac{1}{K_1}+\frac{1}{K_m})\frac{1}{\alpha^2} \quad (1)$$

The ratio of free ammonia to total ammonia in hydrolysate can be calculated by the following formula:

$$\alpha=\frac{[NH_3]}{[NH_3]+[NH_4^+]}=\frac{[OH^-]}{K_b+[OH^-]}=\frac{K_a}{K_a+[H^+]} \quad (2)$$

$$\alpha=\left(1+\frac{10^{-pH}}{10^{-(0.09018+\frac{2729.29}{T(K)})}}\right)^{-1} \quad (3)$$

where, K_a and K_b are the dissociation constants; T is the temperature of the solution; α represents the fraction of free ammonia.

Feed-side resistance (1/K_l):

$$Sh=\frac{K_1 d}{D_{A,W}}=1.62(\frac{d^2 v}{l D_{A,W}})^{0.33} \quad (4)$$

In the equation, Sh represents the Sherwood number; K_l is the mass transfer coefficient, m/s; d is the inner diameter of the membrane tube, l is the membrane length, m; v is the feed flow rate, m/s; $D_{A,W}$ is the diffusion coefficient of ammonia in water, m/s.

Membrane pore resistance (1/K_m):

$$K_m=\frac{\varepsilon D_{A,M} H}{\tau\delta} \quad (5)$$

In the equation, $D_{A,M}$ represents the diffusion coefficient of ammonia in the membrane, m/s; ε is the porosity; τ is the curvature; δ is the membrane thickness; H is the Henry constant.
